# Supplementary material for: The microbiome biomarkers of pregnant women’s vaginal area predict preterm prelabor rupture in Western China
Source: Front Cell Infect Microbiol. 2024 Oct 31;14:1471027. doi: 10.3389/fcimb.2024.1471027 (PMC11560878; doi:10.3389/fcimb.2024.1471027)
Supplement: Supplementary file 1 [file DataSheet1.zip › compare_1/Community/Structure_Barplot/class/class.taxon.Barplot.Legend.With.phylum.pdf]

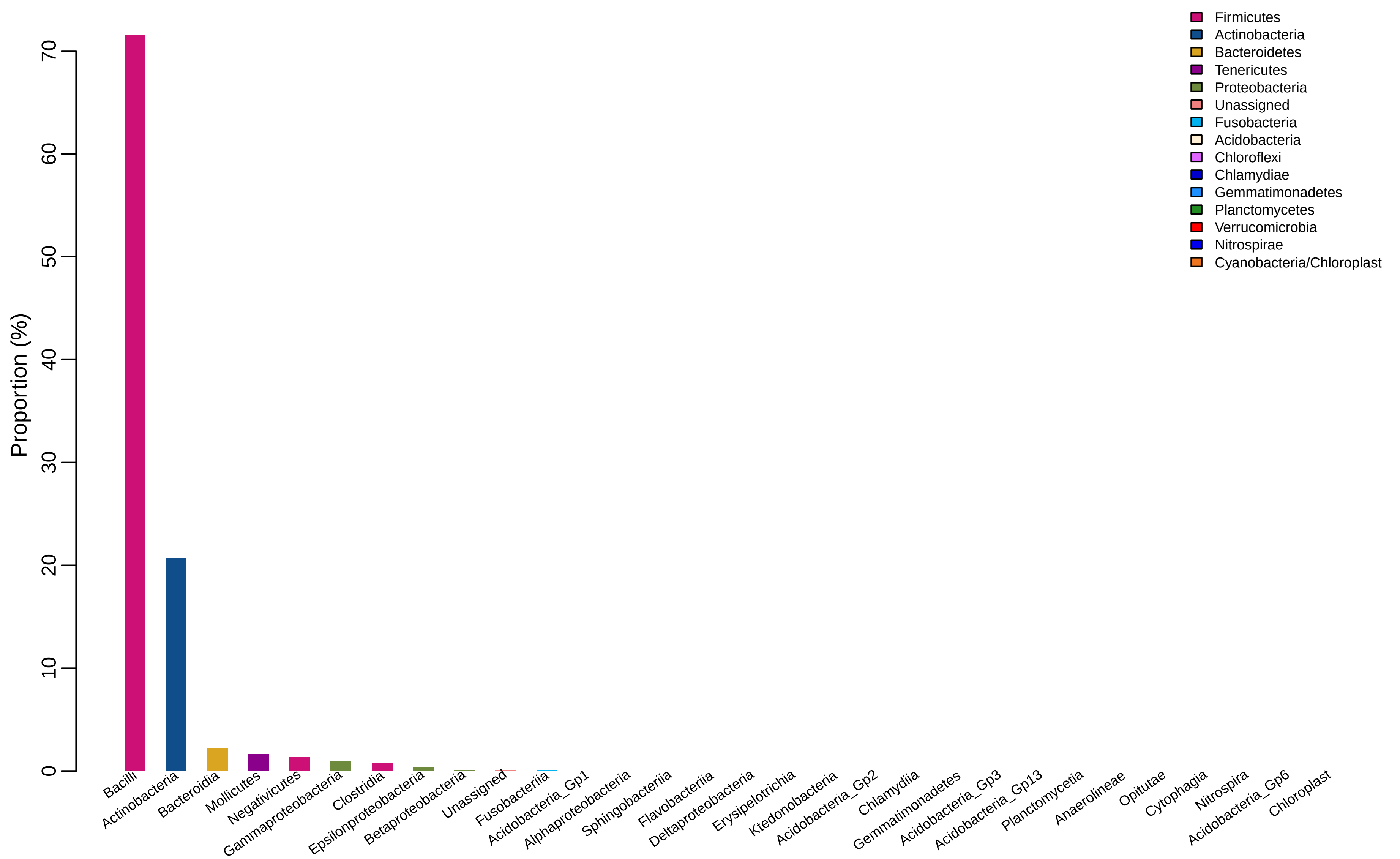
Proportion (%)
0
10
20
30
40
50
60
70
Firmicutes
Actinobacteria
Bacteroidetes
Tenericutes
Proteobacteria
Unassigned
Fusobacteria
Acidobacteria
Chloroflexi
Chlamydiae
Gemmatimonadetes
Planctomycetes
Verrucomicrobia
Nitrospirae
Cyanobacteria/Chloroplast
Bacilli
Actinobacteria
Bacteroidia
Mollicutes
Negativicutes
Gammaproteobacteria
Clostridia
Epsilonproteobacteria
Betaproteobacteria
Unassigned
Fusobacteriia
Acidobacteria_Gp1
Alphaproteobacteria
Sphingobacteriia
Flavobacteriia
Deltaproteobacteria
Erysipelotrichia
Ktedonobacteria
Acidobacteria_Gp2
Chlamydiia
Gemmatimonadetes
Acidobacteria_Gp3
Acidobacteria_Gp13
Planctomycetia
Anaerolineae
Opitutae
Cytophagia
Nitrospira
Acidobacteria_Gp6
Chloroplast
